# Prognostic Value of Glasgow Prognostic Score in Non-small Cell Lung Cancer: A Systematic Review and Meta-Analysis

**DOI:** 10.3389/pore.2022.1610109

**Published:** 2022-02-15

**Authors:** Chuan-Long Zhang, Kui Fan, Meng-Qi Gao, Bo Pang

**Affiliations:** ^1^ International Medical Department of Guang’anmen Hospital, China Academy of Chinese Medical Sciences, Beijing, China; ^2^ Department of Radiation Oncology, Cangzhou Hospital of Integrated TCM-WM Hebei, Cangzhou, China; ^3^ College of Traditional Chinese Medicine, Shandong University of Traditional Chinese Medicine, Jinan, China

**Keywords:** meta-analysis, non-small cell lung cancer, systematic review, prognostic, GPS

## Abstract

**Background:** Systemic inflammation is a key factor in tumor growth. The Glasgow Prognostic Score (GPS) has a certain value in predicting the prognosis of lung cancer. However, these results still do not have a unified direction.

**Methods:** A systematic review and meta-analysis were performed to investigate the relationship between GPS and the prognosis of patients with non-small cell lung cancer (NSCLC). We set patients as follows: GPS = 0 vs. GPS = 1 or 2, GPS = 0 vs. GPS = 1, GPS = 0 vs. GPS = 2. We collected the hazard ratio (HR) and the 95% confidence interval (CI).

**Results:** A total of 21 studies were included, involving 7333 patients. We observed a significant correlation with GPS and poor OS in NSCLC patients (HR_GPS=0 vs. GPS=1 or 2_ = 1.62, 95% CI: 1.27–2.07, *p* ≤ .001; HR_GPS=0 vs GPS=1_ = 2.14, 95% CI:1.31–3.49, *p* ≤ .001; HR_GPS=0 vs. GPS=2_ = 2.64, 95% CI: 1.45–4.82, *p* ≤ .001). Moreover, we made a subgroup analysis of surgery and stage. The results showed that when divided into GPS = 0 group and GPS = 1 or 2 group, the effect of high GPS on OS was more obvious in surgery (HR = 1.79, 95% CI: 1.08–2.97, *p* = .024). When GPS was divided into two groups (GPS = 0 and GPS = 1 or 2), the III-IV stage, higher GPS is associated with poor OS (HR = 1.73, 95% CI: 1.43–2.09, *p* ≤ .001). In the comparison of GPS = 0 and GPS = 1 group (HR = 1.56, 95% CI: 1.05–2.31, *p* = .026) and the grouping of GPS = 0 and GPS = 2(HR = 2.23, 95% CI: 1.17–4.26, *p* = .015), we came to the same conclusion.

**Conclusion:** For patients with NSCLC, higher GPS is associated with poor prognosis, and GPS may be a reliable prognostic indicator. The decrease of GPS after pretreatment may be an effective way to improve the prognosis of NSCLC.

## Introduction

The burden of cancer morbidity and mortality is growing rapidly around the world. The number of new deaths from lung cancer was 1,796,144, accounting for 1/5 (18.0%) of cancer deaths in 2020 (1). Non-small cell lung cancer (NSCLC) is an important histological type of primary bronchogenic carcinoma, which is one of the most common malignant tumors, accounting for more than 80% of the total number of lung cancer cases (2, 3). Therefore, it is very urgent to find some reliable and feasible indicators to evaluate the prognosis of patients with NSCLC, to guide individualized treatment and follow-up programs.

Current studies have shown that immune and nutritional status are highly correlated to the occurrence, progression, and the treatment response of cancer (4–6). Systemic inflammation leads to increased protein decomposition and progressive nutritional decline through catabolism. The inflammation parameter is a strong candidate index to predict the prognosis of cancer. The poor prognosis of patients with malignant tumors is often associated with immune-related systemic inflammatory response and malnutrition. Therefore, in recent years, some prognostic markers based on inflammation and nutrition have been introduced, including Glasgow Prognostic Score (GPS) (7), Modified Glasgow prognosis score (MGPS) (8), C-reactive protein-albumin ratio (CRP/ALB, CAR) (9), Prognostic nutrition index (PNI) (10, 11) and advanced lung cancer inflammation index (ALI) (12, 13) to predict the prognosis and survival of patients with lung cancer.

The GPS, which was first reported by Forrest et al., is used to predict the prognosis of patients with NSCLC. The GPS is based on circulating C-reactive protein (CRP) and serum albumin (ALB) levels. The definition of GPS was shown in Table 1 (14). Many scholars have conducted retrospective and prospective studies on the prognostic value of GPS in patients with NSCLC (7, 14–33). However, due to the difference in research design, sample size, and other influencing factors, the conclusions are not completely consistent, and the way of grouping according to GPS is not uniform. Therefore, we conducted this study to fully clarify the prognostic role of GPS in patients with NSCLC.

**TABLE 1:** Description of the preoperative GPS.

	GPS
CRP ≤10 mg/L and albumin ≥3.5 g/dl	0
CRP ≤10 mg/L and albumin <3.5 g/dl	1
CRP >10 mg/L and albumin ≥3.5 g/dl	1
CRP >10 mg/L and albumin <3.5 g/dl	2

GPS, Glasgow Prognostic Score, CRP C-reactive protein.

## Methods

### Search Strategy

**FIGURE 1:**
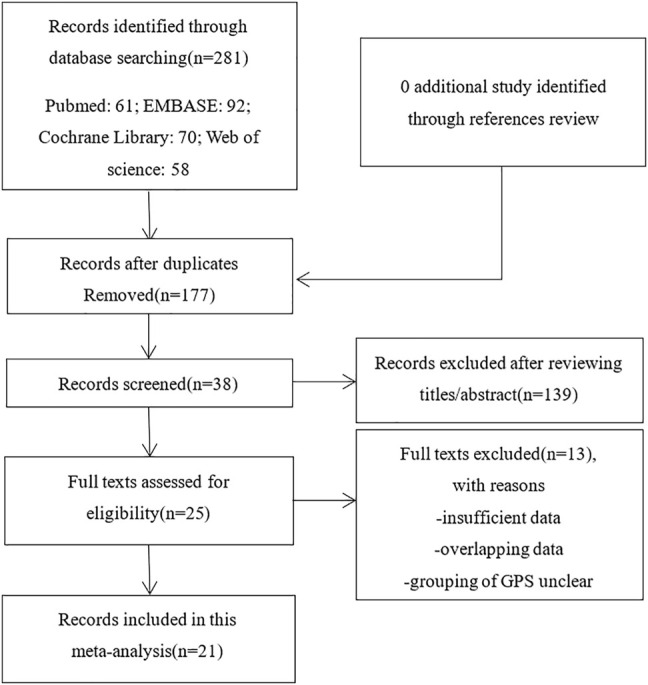
Flow diagram of the study selection process.

We explored the literature databases PubMed, EMBASE, Web of Science, and Cochrane Library for studies that may meet the criteria until April 2021. The search terms were set to “lung adenocarcinoma” OR “Non-Small Cell Lung cancer” OR “NSCLC” OR “LAD” OR “ADC” AND “Glasgow prognostic score”. Determine whether the literature is duplicated by using the author’s name, institution, clinical trial registry number, the number of participants, and baseline data. Among them, if there are studies reported by the same author, the latest and most complete publications would be chosen. Moreover, we manually searched the reference lists describing GPS and patients with NSCLC. The results were limited to humans and the English language. All results were imported into EndNote (Vision X9.2). The selecting process is to be briefed by complying with PRISMA flow diagram (Figure 1) (34).

### Eligibility Criteria

The studies which were included must meet the following criteria: 1) prospective and retrospective study to investigate the prognostic effects of GPS on patients with NSCLC diagnosed by histopathological analysis. 2) the patients were graded strictly according to the definition of GPS(Table 1), and the cases were grouped clearly. 3) publication details were available and complete. 4) the research data provided were sufficient to calculate the hazard ratios (HRs) of the survival rate and its 95% confidence interval (CI). If the HRs cannot be obtained directly from the article, the Kaplan-Meier curve can be calculated (35). 5) the full text was available in English.

If one of the following criteria is met, the study is excluded: 1) reviews, case reports, conference abstracts, chapters of books, editorials, and edited letters or author corrections; 2) studies that cannot be used, such as duplication of data, high similarity of data, poor quality of literature, etc. 3) survival data of studies missing or impossible to calculate; 4) studies of animals. In addition, if the data subset is published in many articles, only the latest articles are included.

### Data Extraction and Quality Assessment

Design a standardized extraction table at first. The characteristics of included studies contented the last name of the first author, publication year, the country of the study, study type, sample size, patient’s age, gender, follow-up period, treatment, lung cancer type, and TNM stage. Two authors (KF and CLZ) independently assess the characteristics of selected studies. If there was disagreement, it would be resolved through discussions with the third researcher (BP). All the included studies were evaluated by Newcastle-Ottawa Scale (NOS) (36). The score of the scale is between 0 and 9. It is defined as a high-quality study when the score is ≥6.

### Statistical Analysis

Pooled HRs and 95% CI is extracted from each study were used as indicators. We used Cochran’s Q test and Higgin’s *I* ^2^ statistics to evaluate the statistical heterogeneity between pooled studies. A 2-tailed *α* level of .05 was set as the threshold for statistical significance. If *p* < .05 and I^2^ > 50%, we will choose the random-effect model in this meta-analysis, otherwise the fixed-effect model will be performed (37). In addition, we have also conducted sensitivity analysis to verify the stability of the results.

Publication bias was evaluated using Begg’s statistical test and Egger’s statistical test. Statistical analysis was performed using STATA version 16.0 (Stata Corporation, College Station, TX, United States).

## Results

### Search Results and Basic Characteristics of the Included Studies

As mentioned above, we searched 281 records in online databases and references. After we deleted duplicates that were not related to GPS, we browsed the full text of the remaining 38 studies. Then, after further qualification evaluation, 25 studies were retained. Of these 25 studies, 2 were first excluded because of data duplication; the other 2 lacked relevant survival data. Finally, 21 studies were included in this analysis after cross-reference. There are no additional studies.

The characteristics of qualified studies are shown in Table 2. In included studies, 13 were conducted in Japan, 4 in China, 3 in the United States, and 1 in Australia. We conducted 21 studies involving a total of 7,333 patients with NSCLC. All 21 studies depicted the association between GPS and OS. Among them, the grouping method of 14 studies is divided into two groups: GPS = 0 and GPS = 1 or 2 (7, 14, 15, 17, 18, 20, 21, 23, 24, 27–29, 32, 33). Seven studies were compared twice, grouped by GPS = 0 and GPS = 1, GPS = 0 and GPS = 2 (16, 19, 22, 25, 26, 30, 31).

**TABLE 2:** The basic characteristics of the included studies.

Author	Year	Country	Study type	Sample size (*N*)	GPS = 0	GPS = 1	GPS = 2	Age (years)	Gender (M/F)	Follow-up (months)	Stage	Treatment	Lung cancer type
Forrest (7)	2003	United Kingdom	PO and RO	161	27	101	33	<60 (37); >60 (124)	105/56	NA	III–IV	Non-surgery (RT + CT)	Squamous (64); Adenocarcinoma (53); Others (44)
Forrest (14)	2004	United Kingdom	PO	109	27	69	13	<60 (41); >60 (68)	63/46	NA	III–IV	Non-surgery (CT)	Squamous (40); Adenocarcinoma (46); Others (23)
Forrest (33)	2005	United Kingdom	PO	101	32	59	10	<60 (18); >60 (83)	62/39	NA	III–IV	Non-surgery (NA)	NA
Miyazaki (29)	2015	Japan	RO	97	65	25	7	>80	62/35	NA	I–IV	Surgery	NA
Fan (28)	2016	China	RO	1745	668	647	430	≤55 (160); >55, ≤70 (754); >70 (831)	1,217/528	20 (median)	I–IV	Non-surgery (CT)	NA
Yotsukura (24)	2016	Japan	RO	1,048	817	184	47	<65 (481); ≥65 (567)	597/451	NA	I–II	Surgery	Squamous (180); Adenocarcinoma (754); Others (114)
Miyazaki (23)	2017	Japan	RO	108	99	4	5	82 (80–93)	69/39	NA	I–IV	Surgery	Adenocarcinoma (76); Others (32)
Tomita (21)	2018	Japan	RO	341	191	112	38	<65 (106); ≥65 (235)	173/168	NA	I–III	Surgery	Adenocarcinoma (268); Others (73)
Kasahara (20)	2019	Japan	RO	47	24	6	17	< 65 (14); ≥65 (33)	37/10	NA	I–IV	Non-surgery (IO)	Squamous (12); Others (35)
Kasahara (18)	2020	Japan	RO	214	141	43	30	<65 (62); ≥65 (152)	83/131	NA	I–IV	Non-surgery (EGFR-TKI)	Adenocarcinoma (212); Others (2)
Takamori (15)	2021	Japan	RO	304	109	85	110	<65 (104); ≥65 (208)	242/62	NA	IIIb–IV	Non-surgery (IO)	Squamous (74); Others (230)
Tomita (32)	2014	Japan	RO	312	264	31	17	<65 (104); ≥65 (208)	192/129	NA	I–III	Surgery	Adenocarcinoma (237); Others (75)
Lindenmann (17)	2020	Australia	PO	300	229	68	3	65.4 ± 10.0 (20–87)	187/113	38.1 ± 28.3	I	Surgery	Squamous (95); Adenocarcinoma (191); Others (14)
Machida (27)	2016	Japan	RO	156	136	16	4	<65 (70); ≥65 (86)	75/81	48.0	IA–IIIA	Surgery	Adenocarcinoma
Kawashima (30)	2015	Japan	RO	1,043	897	107	39	NA	671/372	36.0–60.0	I–III	Surgery	Squamous (220); Adenocarcinoma (741); Others (82)
Jiang (31)	2014	China	PO	138	95	32	11	55 (37–81)	117/21	24.0–60.0	IIIB–IV	Non-surgery (CT)	Squamous (67); Adenocarcinoma (48); Others (23)
Osugi (26)	2016	Japan	RO	327	286	30	11	≤69 (171); >69 (156)	199/128	≥60.0	I–III	Surgery	Squamous (78); Adenocarcinoma (232); Others (17)
Su (25)	2016	China	PO	207	49	111	47	<60 (126); ≥60 (81)	144/63	NA	IIIA–IV	Non-surgery (CT)	Squamous (63); Adenocarcinoma (127); Others (17)
Ni (22)	2018	China	RO	436	NO	NO	NO	≤62 (228); >62 (208)	297/139	NA	III–IV	Non-surgery (RT + CT)	Squamous (107); Others (329)
Topkan (19)	2019	Japan	RO	83	42	22	19	>70	49/34	NA	IIIb	Non-surgery (RT + CT)	Squamous (47); Adenocarcinoma (36)
Kikuchi (16)	2020	Japan	RO	56	31	16	9	71 (65–77)	40/16	NA	III–IV	NA	Squamous (25); Adenocarcinoma (28); Others (3)

GPS, Glasgow Prognostic Score; *N*, numbers of studies; *p*, *p*-values of Q test; NA, not available; PO, prospective studies; RO, retrospective studies; CT, chemo therapy; RT, radiation therapy; IO, immunotherapy; EGFR-TKI, Epidermal growth factor receptor-tyrosine kinase inhibitor.

### Qualitative Assessment

According to the evaluation of the NOS, all the included reports were considered high-quality (Table 3).

**TABLE 3:** Quality assessment based on the NOS.

Study	Year	Selection	Comparability	Outcome	Total score
Forrest (7)	2003	4	2	2	8
Forrest (14)	2004	4	2	2	8
Forrest (33)	2005	4	2	2	8
Miyazaki (29)	2015	4	2	2	8
Fan (28)	2016	4	2	1	7
Yotsukura (24)	2016	4	2	2	8
Miyazaki (23)	2017	4	2	2	8
Tomita (21)	2018	4	2	2	8
Kasahara (20)	2019	4	2	2	8
Kasahara (18)	2020	4	2	2	8
Takamori (15)	2021	4	2	2	8
Tomita (32)	2014	4	2	2	8
Lindenmann (17)	2020	4	2	2	8
Machida (27)	2016	4	2	2	8
Kawashima (30)	2015	4	2	2	8
Jiang (31)	2014	4	2	2	8
Osugi (26)	2016	4	2	2	8
Su (25)	2016	4	2	2	8
Ni (22)	2018	4	2	2	8
Topkan (19)	2019	3	2	2	7
Kikuchi (16)	2020	3	2	2	7

NOS, Newcastle–Ottawa Quality Assessment Scale.

### Meta-Analysis Results

#### Overall Survival

**FIGURE 2:**
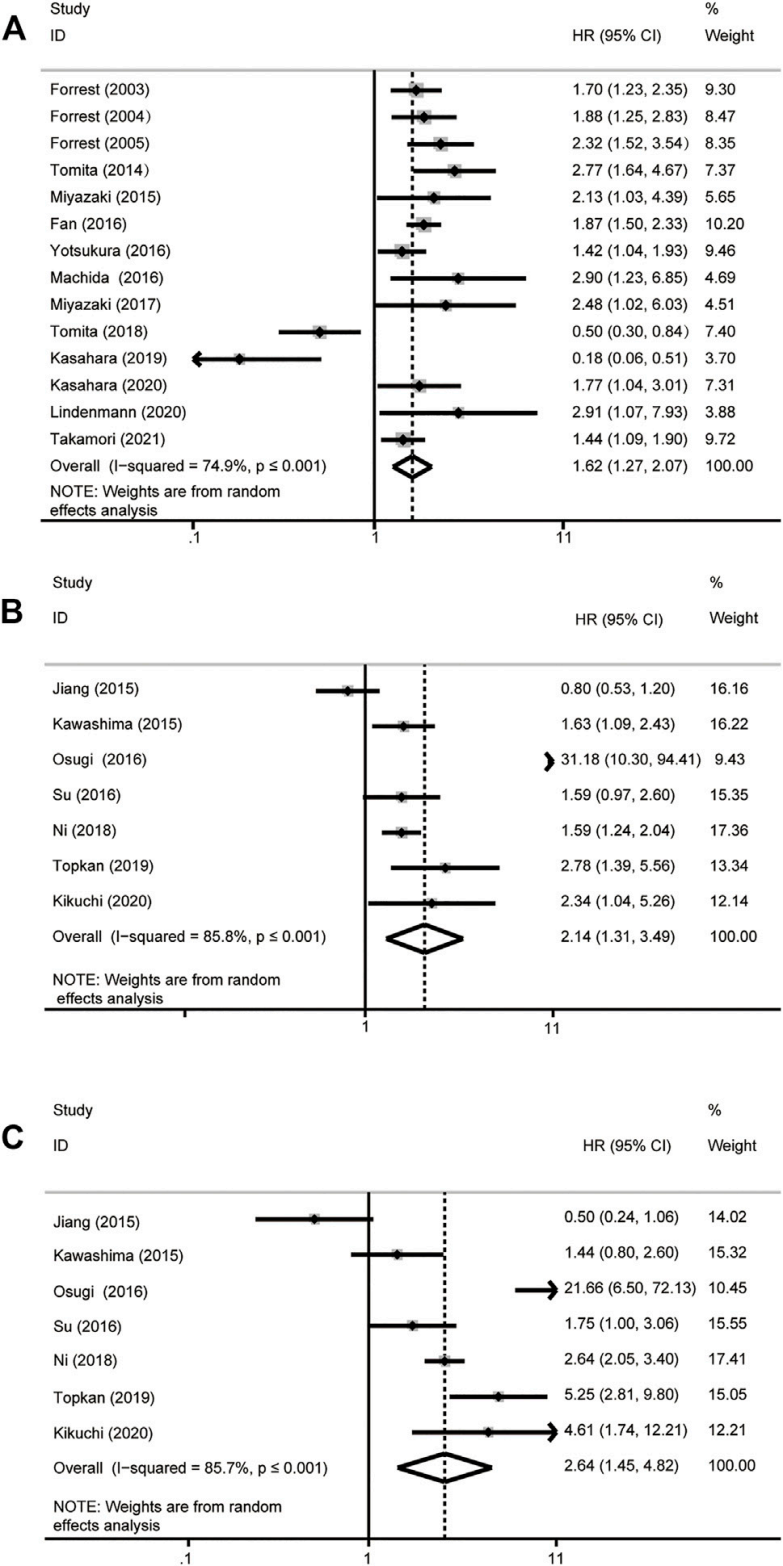
Forest plot of overall survival analysis. **(A)** GPS = 0 vs GPS = 1 or 2. **(B)** GPS = 0 vs GPS = 1. **(C)** GPS = 0 vs GPS = 2. *HR, hazard ratio; 95% CI, 95% confidence interval.*

A total of 21 studies including 7,333 patients were included in the analysis of HR for OS (Supplementary Table S1). We choose the random-effect model (*I* ^2^ > 50%, *p* ≤ .001). The results showed that higher GPS is associated with poor OS in patients with NSCLC. The grouping method of 14 studies is divided into two groups: GPS = 0 and GPS = 1 or 2, of which results showed that there is a significant correlation between GPS and OS (HR = 1.62, 95% CI: 1.27–2.07, *p* ≤ .001) (Figure 2A). The results of the other 7 studies revealed that higher GPS was related to the poor OS (HR_GPS=0 vs. GPS=1_ = 2.14, 95% CI: 1.31–3.49, *p* ≤ .001; HR_GPS=0 vs. GPS=2_ = 2.64, 95% CI: 1.45–4.82, *p* ≤ .001) (Figures 2B,C).

#### Subgroup Analyses

Furthermore, subgroup analysis was performed according to whether or not surgery and different stages to detect the prognostic value of GPS in patients with NSCLC. We found that when the GPS = 0 group was compared with the GPS = 1 or 2 group, the effect of high GPS on OS was more significant in surgery patients (HR = 1.79, 95% CI: 1.08–2.97, *p* = .024). However, the influence of high GPS on OS of surgical patients was more significant (HR_GPS=0 vs GPS=1_ = 6.80, 95% CI: .38–122.45; HR_GPS=0 vs. GPS=2_ = 5.31, 95% CI: .37–75.45), the result compared with the non-surgery group was not statistically significant (P_GPS=0 vs. GPS=1_ = .194; P_GPS=0 vs. GPS=2_ = .218). After the subgroup analysis of the stage, for patients with NSCLC, we found that when the GPS = 0 group was compared with the GPS = 1 or 2 group, the effect of high GPS on poor OS was the most obvious in the III-IV stage (HR = 1.73, 95% CI: 1.43–2.09, *p* ≤ .001) than in other stages. In the comparison of GPS = 0 and GPS = 1 group (HR = 1.56, 95% CI: 1.05–2.31, *p* = .026) and the grouping of GPS = 0 and GPS = 2(HR = 2.23, 95% CI: 1.17–4.26, *p* = .015), we came to the same conclusion. However, there was no significance during the I-III period when the GPS = 0 group was compared with the GPS = 1 group (*p* = .194) or the GPS = 0 group was compared with the GPS = 2 group (*p* = .218) (Table 4). Therefore, we consider that the stage of NSCLC and whether or not surgery may be the source of heterogeneity.

**TABLE 4:** The subgroup analysis according to whether or not surgery and different stages.

Group	Analysis	*N*	References	Random-effects model		Heterogeneity	
				HR (95%CI)	*p*	*I* ^*2*^ (%)	*p*
GPS = 0 vs. GPS = 1 or 2	Subgroup 1						
	Surgery	7	(17, 21, 23, 24, 27, 29, 32)	1.79 (1.08–2.97)	.024	79.00	≤.001
	Non-surgery	7	(7, 14, 15, 18, 20, 28, 33)	1.59 (1.21–2.10)	≤.001	73.20	≤.001
	Subgroup 2						
	Stage I–II	2	(17, 24)	1.72 (.92–3.22)	.087	44.50	.180
	Stage I–III	3	(21, 27, 32)	1.56 (.45–5.35)	.482	91.80	≤.001
	Stage III–IV	4	(7, 14, 15, 33)	1.73 (1.43–2.09)	≤.001	18.40	.299
	Stage I–IV	5	(18, 20, 23, 28, 29)	1.41 (.78–2.52)	.251	79.60	≤.001
GPS = 0 vs GPS = 1	Subgroup 1						
	Surgery	2	(26, 30)	6.80 (.38–122.45)	.194	95.90	≤.001
	Non-surgery	4	(19, 22, 25, 31)	1.47 (.95–2.26)	.082	75.60	.006
	Subgroup 2						
	Stage I–III	2	(26, 30)	6.80 (.38–122.45)	.194	95.90	.009
	Stage III–IV	5	(16, 19, 22, 25, 31)	1.56 (1.05–2.31)	.026	70.70	≤.001
GPS = 0 vs GPS = 2	Subgroup 1						
	Surgery	2	(26, 30)	5.31 (.37–75.45)	.218	95.10	≤.001
	Non-surgery	4	(19, 22, 25, 31)	1.83 (.73–4.57)	.195	94.70	≤.001
	Subgroup 2						
	Stage I–III	2	(26, 30)	5.30 (.37–75.32)	.218	93.60	≤.001
	Stage III–IV	5	(16, 19, 22, 25, 31)	2.23 (1.17–4.26)	.015	84.70	≤.001

GPS, Glasgow Prognostic Score; *N*, number of studies; HR, hazard ratio; 95% CI, 95% confidence interval, *p*, *p*-values of Q test; OS, overall survival; VS, versus.

## Sensitivity Analysis

**FIGURE 3:**
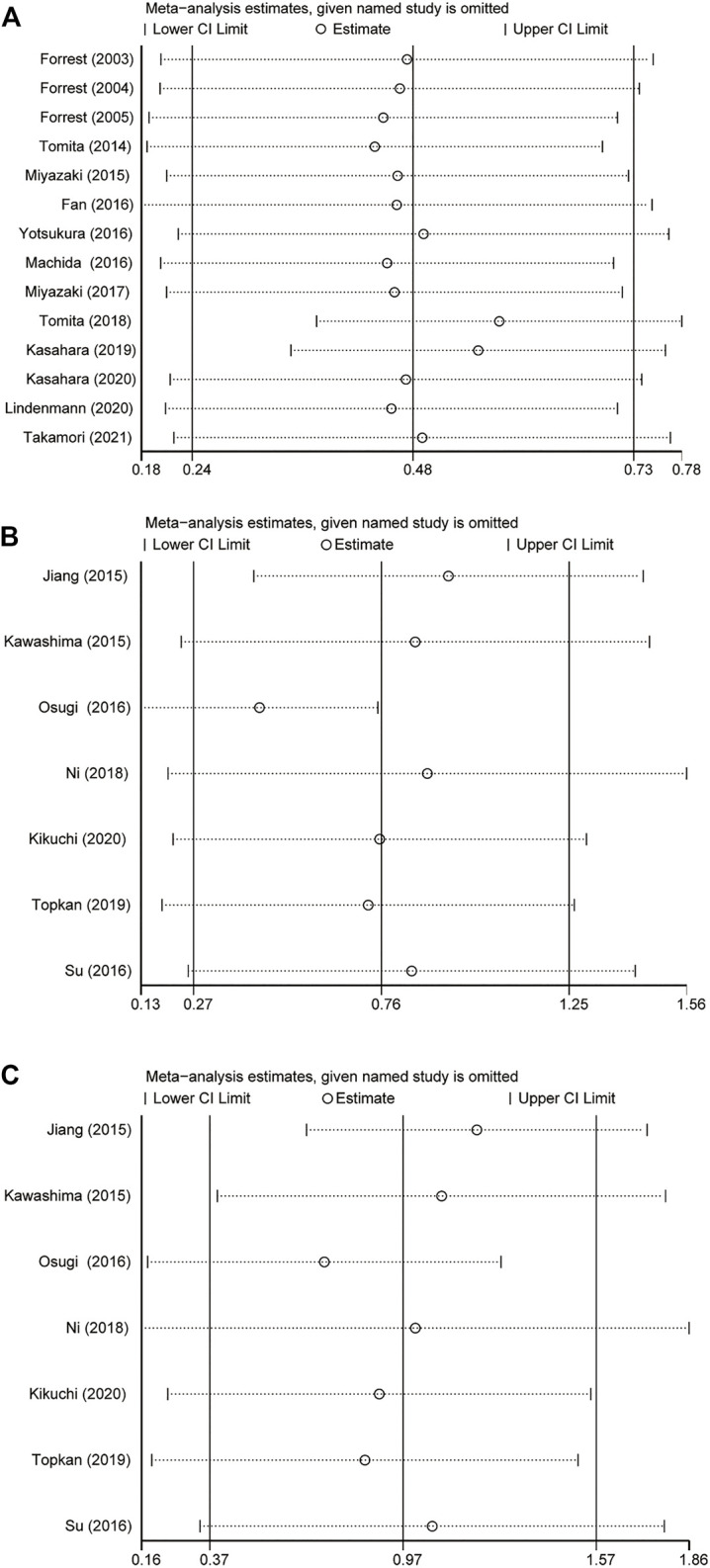
Result of sensitivity analyses by omitting one study in each turn. **(A)** GPS = 0 vs GPS = 1 or 2. **(B)** GPS = 0 vs GPS = 1. **(C)** GPS = 0 vs GPS = 2. GPS, *Glasgow Prognostic Score; 95% CI, 95% Confidence Interval.*

The results showed that excluding any single literature had no significant effect on the collection of HR after sensitivity analysis of 21 studies. This shows that our analysis results were robust (Figures 3A–C).

### Publication Bias Assessment

**FIGURE 4:**
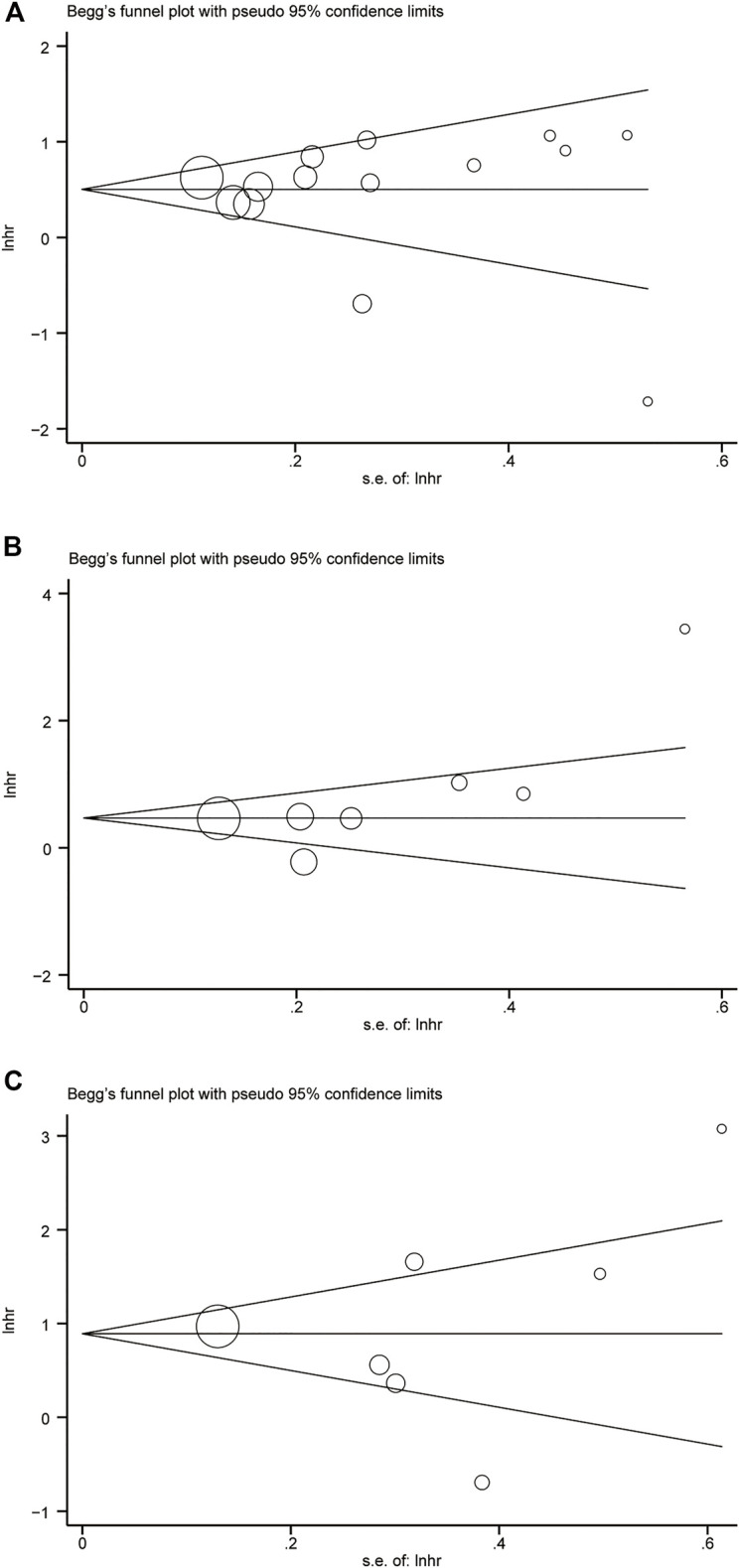
Begg’s funnel plot. **(A)** GPS = 0 vs GPS = 1 or 2. **(B)** GPS = 0 vs GPS = 1. **(C)** GPS = 0 vs GPS = 2.

Considering the risk of bias may affect the results of meta-analysis, assessment of potential publication bias using Begg’s funnel chart and Egger’s test. The results showed that the two methods did not produce bias, which proves the reliability of the results (Figures 4A–C).

## Discussion

NSCLC as a kind of cancer with high morbidity and mortality seriously endangers people’s health and quality of life. At present, there is more and more evidence that systemic inflammatory response and systemic immune response defects play an important role in cancer invasion and progression (38). Although inflammation-related prognostic indicators have received some attention in NSCLC, the mechanism of the survival relationship between them is not clear, which may be related to malnutrition, immunodeficiency, up-regulation of growth factors, or angiogenesis.

CRP is a representative acute phase reaction, its level increases rapidly in inflammation, and is considered to be one of the most widely used indicators of systemic inflammation. Many studies have proved that CRP plays an important role in the diagnosis and prognosis of NSCLC (39–42). ALB is the most commonly used to evaluate nutritional status, and low ALB in patients with NSCLC usually indicates weight loss and malnutrition (43–45). In addition, as early as 2001, Mcmillan found that the increase of CRP concentration in circulation was always accompanied by the decrease of ALB concentration (46). Therefore, systemic inflammation may affect the concentration of serum ALB. The relationship between CRP and ALB was proposed by Forrest et al. For the first time, they combined CRP and serum ALB as prognostic scores, and confirmed their prognostic value in patients with NSCLC (13), which was defined as Glasgow prognostic score (47). Gradually, the value of GPS in predicting prognosis has been confirmed in many studies.

High GPS is highly related to the poor prognosis of many different types of tumors, but its value in the prognosis of patients with NSCLC is still contentious (15, 16). This study was a relatively comprehensive meta-analysis to investigate the value of GPS in predicting the prognosis for patients with NSCLC. In this study, we performed a meta-analysis including 21 studies with a total of 7,333 patients. As far as we know, this is the first meta-analysis that the GPS group divided into GPS = 0 with GPS = 1 or 2, GPS = 1 with GPS = 2, and GPS = 0 with GPS = 2. The OS of patients with NSCLC was evaluated by comparing the HRs between different groups to explore the relationship between GPS and OS. In addition, we also conducted a subgroup analysis of treatment and stage, which better demonstrated the prognostic value of GPS in patients with NSCLC.

The results of the study showed that high GPS predicted a poor OS in patients with NSCLC. Subgroup analysis was according to surgery and stage showed that GPS = 1 or 2 was more likely to predict poor OS in patients undergoing surgery. Moreover, based on the results of subgroup analysis of stage, we had reason to believe that the prognostic value of GPS was more significant in NSCLC patients with III-IV stage. When GPS = 1 or 2, patients undergoing surgery would face worse OS than patients without surgery, suggesting that clinicians should pay attention to the inflammatory status and nutritional status of patients during treatment. This is also the biggest difference between our study and Jin et al. (48), whose study is the antecedent of our study and shows that the association between MGPS and poor OS is not significant in patients undergoing surgery. This difference may be due to the fact that some patients with GPS = 1 are included in patients with MGPS = 0. For patients with NSCLC who have undergone surgery, the use of GPS to predict prognosis may be more sensitive than the use of MGPS to evaluate. This is worthy of further study. Anyway, controlling inflammation and improving nutritional status as far as possible is one of the key measures to ensure a good prognosis of patients with NSCLC.

Our study has certain limitations. Firstly, Japanese and Chinese studies accounted for the vast majority of included studies, which led to selection bias. Secondly, in the literature selection, we only chose the research that could obtain the full text in English. This may lead to language bias. Thirdly, there were many differences in measuring CRP and ALB levels, such as the time, place, method, and personnel of the measurement. However, sensitivity analysis showed that the results of this meta-analysis were reliable at least. Finally, since only 4 studies reported the prognostic role of progress-free survival (PFS), we did not analyze PFS, which meant that there were limitations in the selection of prognostic indicators. Therefore, in our meta-analysis, potential heterogeneity may be inevitable. Well-designed studies and repeated measurements in a larger population may help to evaluate the prognostic value and other clinical significance of GPS in NSCLC.

## Conclusion

For patients with NSCLC, higher GPS is associated with a poor prognosis. GPS is an independent risk factor for OS and maybe a reliable prognostic indicator in NSCLC. The decrease of GPS after pretreatment may be an effective way to improve the prognosis of NSCLC.

## Data Availability

The original contributions presented in the study are included in the article/Supplementary Material, further inquiries can be directed to the corresponding author.
